# Distinct Metabolic Requirements of Exhausted and Functional Virus-Specific CD8 T Cells in the Same Host

**DOI:** 10.1016/j.celrep.2016.06.078

**Published:** 2016-07-21

**Authors:** Anna Schurich, Laura J. Pallett, Danyal Jajbhay, Jessica Wijngaarden, Itziar Otano, Upkar S. Gill, Navjyot Hansi, Patrick T. Kennedy, Eleni Nastouli, Richard Gilson, Christian Frezza, Sian M. Henson, Mala K. Maini

**Affiliations:** 1 Division of Infection and Immunity, University College London, London WC1E 6JF, UK; 2 Hepatology Unit, Centre for Immunobiology, Blizard Institute, Barts and The London School of Medicine and Dentistry, Queen Mary University of London, London E1 2AT, UK; 3 Department of Clinical Virology, University College London Hospital, London WC1N 1EH, UK; 4 Institute of Child Health, University College London, London WC1N 1EH, UK; 5 Research Department of Infection and Population Health, University College London, London WC1E 6JB, UK; 6 MRC Cancer Unit, University of Cambridge, Hutchison/MRC Research Centre, Box 197, Cambridge Biomedical Campus, Cambridge CB2 0XZ, UK; 7 William Harvey Research Institute, Barts and the London School of Medicine and Dentistry, Queen Mary University of London, Charterhouse Square, London EC1M 6BQ, UK

## Abstract

T cells undergo profound metabolic changes to meet the increased energy demands of maintaining an antiviral response. We postulated that differences in metabolic reprogramming would shape the efficacy of CD8 T cells mounted against persistent viral infections. We found that the poorly functional PD-1^hi^ T cell response against hepatitis B virus (HBV) had upregulated the glucose transporter, Glut1, an effect recapitulated by oxygen deprivation to mimic the intrahepatic environment. Glut1^hi^ HBV-specific T cells were dependent on glucose supplies, unlike the more functional cytomegalovirus (CMV)-specific T cells that could utilize oxidative phosphorylation in the absence of glucose. The inability of HBV-specific T cells to switch to oxidative phosphorylation was accompanied by increased mitochondrial size and lower mitochondrial potential, indicative of mitochondrial dysfunction. Interleukin (IL)-12, which recovers HBV-specific T cell effector function, increased their mitochondrial potential and reduced their dependence on glycolysis. Our findings suggest that mitochondrial defects limit the metabolic plasticity of exhausted HBV-specific T cells.

## Highlights

• Exhausted CD8 T cells show increased expression of glucose transporter-1
• Functional, but not exhausted, T cells can use OXPHOS to supplement their energy demand
• Exhausted T cells harbor dysfunctional depolarized mitochondria
• Interleukin-12 can rescue mitochondrial function and effector responses in exhausted CD8

## Graphical Abstract

**Figure:**
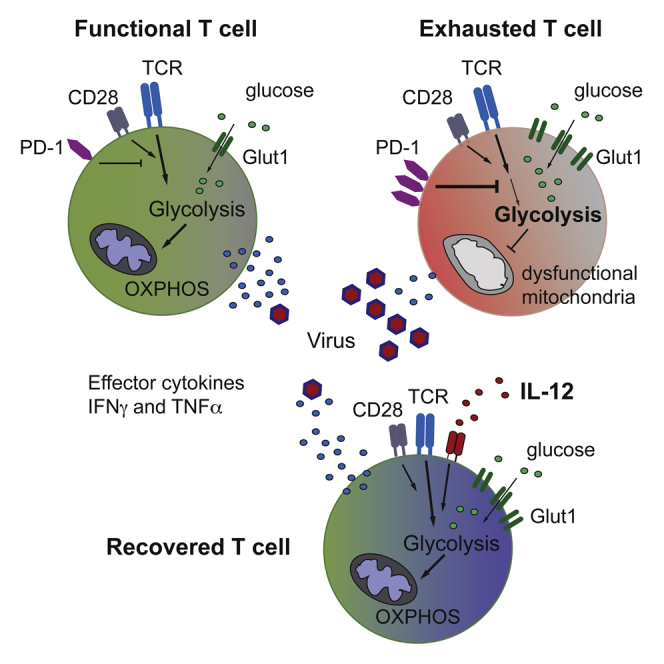

## Teaser

T cells undergo extensive metabolic changes upon activation. Schurich et al. find that functional and exhausted human-virus-specific CD8 T cells have distinct metabolic phenotypes, shaping their effector capacity.

## Introduction

On average, humans are infected with around 8–12 different persistent viruses during their lifetime (Virgin et al., 2009). Most of these infections, like Epstein-Barr Virus (EBV) and cytomegalovirus (CMV), are benign in the vast majority of human hosts, and the antiviral T cell response is adapted to keeping the virus at bay while limiting organ damage. Other chronic infections, such as HIV, hepatitis C virus (HCV), and hepatitis B virus (HBV), cannot be controlled by the T cell response once persistence is established, often resulting in immunopathology and serious sequelae.

An estimated 240 million people worldwide are chronically infected with HBV, which is the leading cause of liver cirrhosis and hepatocellular carcinoma. CD8 T cells are one of the critical mediators of HBV clearance, by interferon (IFN)γ-mediated non-cytopathic mechanisms, possibly supported by direct cytotoxicity. However in chronic HBV infection, the pivotal anti-viral CD8 T cell response is virtually absent. The few HBV-specific T cells detectable are functionally exhausted, with expression of multiple co-inhibitory receptors and poor effector function (Ferrari, 2015), a state that has recently been suggested to allow them to adapt to the onslaught of high-dose antigen (Staron et al., 2014, Utzschneider et al., 2013).

In contrast, T cells directed against CMV are a prototype of a functional response able to efficiently contain this highly prevalent, persistent viral infection. CMV-specific T cells can readily be detected in greatly expanded numbers, with conserved clonotypes often dominating the endogenous T cell repertoire (Khan et al., 2002). They are phenotypically distinct, expressing late differentiation markers such as KLRG-1 rather than the multiple co-inhibitory receptors characteristic of HBV-specific T cells (Schurich and Henson, 2014). CMV-specific T cells produce significant amounts of effector cytokines such as IFNγ and tumor necrosis factor (TNF) in response to stimulation with their cognate peptide in vitro.

Since HBV- and CMV-specific T cells are both directed against persistent viruses but differ markedly in their functionality and phenotype, we were interested in comparing their underlying metabolic requirements. It is increasingly recognized that adequate nutrient supply and energy production are key determinants of the capacity of T cells to proliferate and mediate effector function (Pearce and Pearce, 2013). Naive and resting T cells make use of fatty acid oxidation and the mitochondrial tricarboxylic acid (TCA) cycle, which provides reducing agents for energy production through oxidative phosphorylation (OXPHOS) (Pearce et al., 2009). Recently, it has been shown in murine models that mitochondrial activity is also needed for activating and maintaining antigen-specific responses (Okoye et al., 2015, Sena et al., 2013). Upon activation, CD8 T cells have been described to switch their metabolism to become heavily dependent on glycolysis, even in the presence of sufficient oxygen. Despite being less energy efficient, glycolysis provides fast energy and metabolites to support proliferation and effector function (MacIver et al., 2013).

Many recent advances in the understanding of T cell metabolism in naive, effector, and memory stages have been made (Pearce and Pearce, 2013). However, the current knowledge of T cell metabolism in chronic viral infections is essentially limited to a single example, the murine model of LCMV (lymphocytic choriomeningitis virus) (Schurich and Henson, 2014). Here, we examine the metabolic requirements and restrictions of exhausted HBV-specific CD8 T cells to the more functional CMV-specific T cells within the same patients. Our data show that CD8 T cells specific for these two chronic viral infections have distinct metabolic profiles. CMV-specific T cells can fuel their energetic demands by making use of both glycolysis and OXPHOS to exert full effector functions. In contrast, exhausted HBV-specific T cells show an impaired capacity to utilize mitochondrial energy supply (OXPHOS), causing a dependence on glycolysis. Their defect in mitochondrial metabolism is rescued by the pro-inflammatory cytokine interleukin (IL)-12, which can stimulate a recovery in HBV-specific effector function (Schurich et al., 2013). Our data suggest that full effector function in human-virus-specific CD8 T cells is dependent on energy supply through both OXPHOS and glycolysis.

## Results

### Glut1 Expression Is Higher in CD8 T Cells Directed against HBV Than CMV

**Figure 1:**
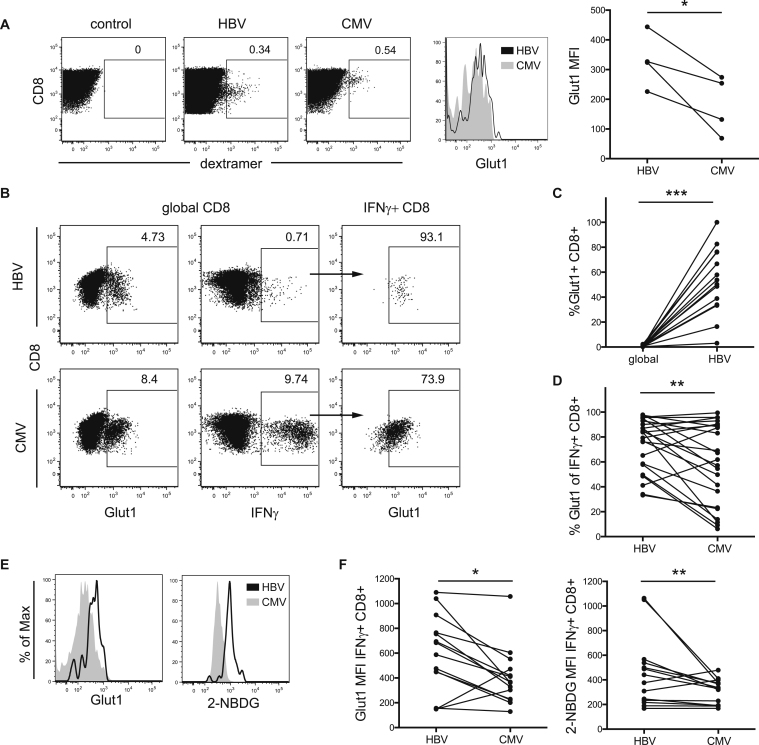
Increased Expression of Glut-1 on HBV-Specific Compared to CMV-Specific CD8 T Cells (A) Glut1 expression in HBV- and CMV-specific T cells after 4-hr stimulation with cognate peptide directly ex vivo. Example of virus-specific cells detected by staining with HLA-A2^+^ dextramers loaded with virus-specific or irrelevant control peptides (left), overlay of Glut1 MFI in HBV- and CMV-specific cells (middle), and summary data (right). (B) Glut1 expression in global CD8 T cells (left), IFNγ response upon culture with HBV- or CMV-specific peptides (middle), and Glut1 expression on virus-specific IFNγ CD8 T cells (right). (C and D) Summary data comparing percentage of Glut1^+^ T cells in global and HBV-specific CD8 T cells (C) and HBV- and CMV-specific T cells (D) in paired samples. (E and F) Comparison of Glut1 expression and 2-NBDG uptake in IFNγ+ HBV- and CMV-specific CD8 T cells, example (E) and summary data (F). ^∗^p < 0.05; ^∗∗^p < 0.005; ^∗∗∗^p < 0.0005.

Upon TCR (T cell receptor) stimulation, T cells markedly increase the expression of de-novo-synthesized glucose transporter 1 (Glut1) to facilitate glucose uptake; this correlates with an increase in glycolysis (Pearce and Pearce, 2013). To examine the capacity for glucose uptake by human-virus-specific CD8 T cells, we analyzed their expression of Glut1 upon activation. First, we stimulated T cells from chronically co-infected patients with HBV- or CMV-derived peptides for 4 hr directly ex vivo and measured Glut1 expression on dextramer-positive T cells. Glut1 expression was higher in activated virus-specific than in global CD8 T cells (data not shown). Glut1 expression was increased in HBV- compared to CMV-specific T cells (Figure 1A), suggesting that a defect in glucose uptake was unlikely to be responsible for their defective cytokine production (Figure S1A).

Since HBV-specific T cells from the circulation can only be detected at very low frequency, we next expanded T cells in culture to study their metabolic phenotype in more detail. We confirmed that the differences observed ex vivo were maintained in vitro. HBV- or CMV-specific CD8 cells were stimulated with their cognate peptides and, upon restimulation, identified by their production of IFNγ and co-stained for Glut1. Again, the frequency of Glut1 expression in virus-specific T cells was significantly increased above the amount expressed by the global CD8 cells in the same sample, confirming that Glut1 is upregulated upon antigen-specific activation (Figures 1B and 1C).

We then compared paired HBV- and CMV-specific CD8 T cell responses from the same donors, which had been activated to comparable levels as assessed by CD38 expression (Figure S1B). The frequency of Glut1-positive cells was, again, higher for HBV-specific than for CMV-specific CD8 in these paired samples (Figures 1B and 1D). We observed the same increased expression of Glut1 in HBV compared to CMV, when cultured T cells were detected by dextramer staining in three patients with known responses to both viruses (Figure S1C).

To confirm that increased Glut1 expression was mediating increased glucose uptake, we pulsed virus-specific T cells with the fluorescent glucose analog 2-NBDG at the end of culture. Increased expression of Glut1 was accompanied by an increase in the uptake of 2-NBDG (Figures 1E and 1F; correlation in Figure S1D).

### Glut1 Upregulation Is Associated with T Cell Exhaustion and Can Be Promoted by Hypoxic Conditions Mimicking the Hepatic Milieu

**Figure 2:**
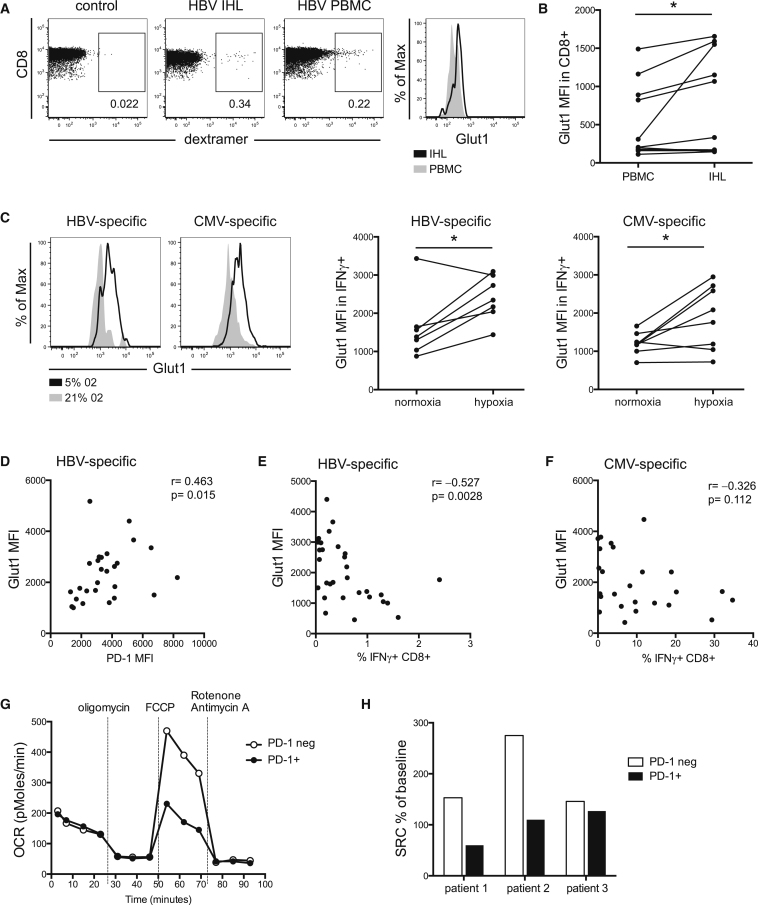
CD8 T Cell Glut1 Expression Can Be Induced by Hypoxia and Correlates with an Exhausted Phenotype (A) Glut1 expression in HBV-specific CD8 T cells, detected by HLA-A2 dextramer staining in paired PBMC and intrahepatic lymphocytes (IHL) directly ex vivo, gated for CD3+CD8+CD4- T cells. (B) Summary data of Glut1 expression on global CD8 T cells from PBMCs and IHL. (C) PBMCs were cultured and restimulated with specific peptides on day 10 for 16 hr in hypoxic (5% O_2_) or normoxic (21% O_2_) conditions to analyze Glut1 mean fluorescence intensity (MFI) in HBV- and CMV-specific T cells. (D–F) Glut1 MFI was plotted against expression of (D) co-inhibitory PD-1, (E) percent IFNγ+ HBV-specific T cells, and (F) percent IFNγ+ CMV-specific T cells. (G) Oxygen consumption rates (OCRs) of purified patient-derived PD-1+ and PD-1− CD8 T cells were measured in real-time ex vivo. Cells were stimulated with anti-CD3 and IL-2 at the time of analysis, and mitochondrial inhibitors were added as indicated (oligomycin is used to block mitochondrial complex V, indicating the amount of oxygen utilized for ATP synthesis; FCCP uncouples ATP synthesis from the electron transport chain by transporting electrons across the inner mitochondrial membrane, allowing the calculation of the spare respiratory capacity; and rotenone/antimycin A finally shut down mitochondrial respiration by blocking complexes I and III). neg, negative. (H) The spare respiratory capacity (SRC) was calculated as percent change in mean baseline OCR to mean maximal OCR after the addition of FCCP in the three patients tested in two independent experiments. ^∗^p < 0.05.

Since the increased Glut1 expression and glucose uptake on poorly functional HBV-specific CD8 was paradoxical, we postulated that it might relate to their site of antigen encounter in vivo. HBV only replicates in the liver, an immune suppressive environment where T cells have restricted supplies of oxygen (Jungermann and Kietzmann, 2000). Glut1 expression was, indeed, increased in paired patient-derived CD8 T cells from liver biopsies compared to PBMCs (peripheral blood mononuclear cells) directly ex vivo. In one HLA-A2^+^ patient, we detected HBV-specific CD8 T cells, confirming increased Glut1 in the liver compared to the periphery (Figures 2A and 2B). Next, we recapitulated the hypoxic environment in vitro by stimulating HBV- and CMV-specific T cells in normoxic versus hypoxic conditions (5% oxygen to mimic the concentration in the hepatic circulation; Jungermann and Kietzmann, 2000). Glut1 expression was promoted in hypoxic conditions (Figure 2C), an effect that was independent of the amount of T cell proliferation (Figure S2A). It is, therefore, possible that a phenotype favoring glycolysis might represent a feedback response promoted by the hepatic environment in which T cells encounter their antigen.

A hallmark of exhaustion is the co-inhibitory receptor PD-1 (Wherry, 2011), which is highly expressed on HBV-specific T cells (Ferrari, 2015). We found that, even during culture, HBV-specific CD8 maintained higher levels of PD-1, accompanied by lower expansion and reduced production of IFNγ+, than CMV-specific CD8 (Figures S2B and S2C) (Schurich et al., 2013). This is consistent with recent work showing that the exhausted phenotype is stable (Utzschneider et al., 2013).

Antigen-stimulated HBV responses showed a correlation between their expression of PD-1 and Glut1 (Figure 2D), suggesting that exhausted T cells increase Glut1 expression. Furthermore, we found a significant inverse correlation between Glut1 expression and the magnitude of T cell expansion, so that HBV-specific cells from patients with the lowest frequency responses expressed the highest levels of Glut1 (Figure 2E). This did not apply to CMV-specific T cells, for which there was no significant correlation between Glut1 and magnitude of response in culture (Figure 2F).

To further probe the metabolic phenotype of patient-derived T cells, we measured cellular oxygen consumption rates (OCRs) during a mitochondrial stress test. The extremely low circulating frequency of HBV-specific T cells precluded their use for this type of biochemical analysis. PD-1 is expressed on recently activated, and maintained on exhausted, CD8 T cells in chronic viral infections (Barber et al., 2005), including HBV (Boni et al., 2007, Fisicaro et al., 2010, Schurich et al., 2013). Studying the impact of PD-1 on global CD8 T cell metabolism in patients with persistent viral infections is important, since therapeutic blockade of this pathway (currently considered in HBV following recent successes in cancer) will affect all PD-1+ cells. Therefore, we sorted PD-1+ and PD-1− CD8 T cells and found that both fractions utilized glycolysis and OXPHOS—as measured, respectively, by their extracellular acidification rate (ECAR) and OCR—to varying degrees in the three patients tested (Figure S2D). However, the spare respiratory capacity (SRC), calculated as percent change in mean OCR at baseline to maximal OCR upon treatment with FCCP (fluorocarbonyl cyanide phenylhydrazone), was lower in PD-1+ compared to PD-1− CD8 T cells (Figures 2G and 2H), a feature retained when cultured in vitro (Figure S2E). SRC is a measure of maximal mitochondrial capacity available to a cell (Henson et al., 2014); therefore, the low SRC suggests that PD-1+ T cells are less well equipped to function in conditions of increased energy demand. The low SRC in PD-1+ cells was at odds with the higher abundance of memory cells in this population (Figure S2F), described to be high utilizers of OXPHOS (van der Windt et al., 2012). Our findings, therefore, reinforced the potential relevance of PD-1 expression, rather than T cell differentiation state, as a driver of the metabolic changes observed.

### HBV-Specific T Cells Are Dependent on Glycolysis for Immediate Effector Function

To further examine the paradoxical increase in glucose uptake by exhausted HBV-specific T cells, we tested their dependence on glycolysis to provide energy. To this end, we stimulated T cells in media containing galactose instead of glucose to prevent them efficiently utilizing glycolysis (Chang et al., 2013). After expansion in regular media, cells were split into either glucose- or galactose-containing media to assess the requirement for glycolysis for immediate effector function upon antigenic restimulation.

**Figure 3:**
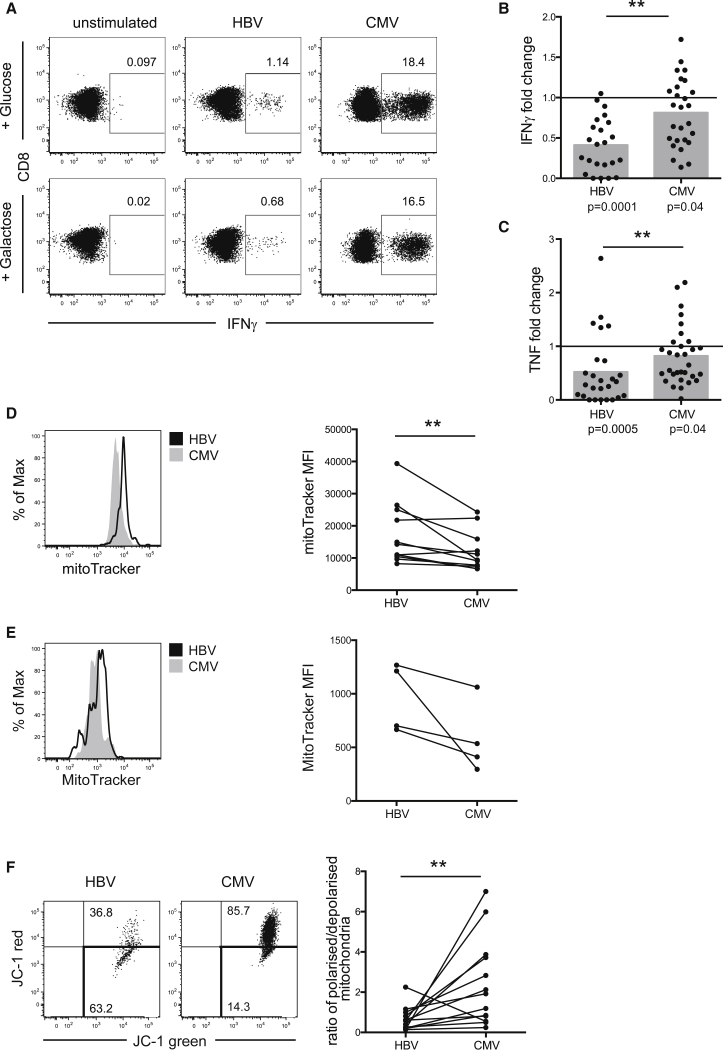
CMV-Specific, but Not HBV-Specific, CD8 T Cells Can Maintain Effector Cytokine Production when Glucose Is Withdrawn (A–C) We cultured PBMCs in complete T cell media before transferring them into media containing either 10 mM glucose or 10 mM galactose for 24 hr before restimulation. Representative example (A) and summary data showing the magnitude of the virus-specific (B) IFNγ and (C) TNF response in galactose, plotted as fold reduction compared to response in glucose (set to 1, indicated as a line in the graphs). Mean response is indicated with gray bars, and individual responses are indicated with dots. (D and E) Comparison of MM in cultured HBV- and CMV-specific T cells by MitoTracker Green staining, representative histograms, and summary data (D) and in dextramer+ virus-specific T cells ex vivo after 4-hr stimulation with cognate peptide; representative histograms and summary data (E). (F) Determination of mitochondrial polarization state by staining with the ratiometric dye JC-1. Red JC-1 staining indicates polarized mitochondria, while loss of red fluorescence shows depolarization. Example fluorescence-activated cell sorting (FACS) plot and comparison of the ratio of polarized/depolarized mitochondria in HBV- and CMV-specific T cells from the same patients. ^∗∗^p < 0.005.

CMV-specific cells showed a diverse response to glucose deprivation, with IFNγ production in some patients being completely unaffected, or even increased, and in others being decreased (Figures 3A and B). Overall, the frequency of IFNγ+ CMV-specific CD8 in the presence of galactose, compared to glucose, was slightly reduced, while, the amount of IFNγ produced per cell was unaffected (Figure S3A). In contrast, the IFNγ response of HBV-specific cells declined strikingly upon culture in galactose (Figures 3A and 3B), with IFNγ MFI (mean fluorescence intensity) also decreasing (Figure S3A). Thus, HBV-specific CD8 showed significantly more dependence on glycolysis than CMV-specific CD8 when mounting an immediate effector response (Figure 3B). Similarly, the frequency of T cells able to produce TNF upon peptide restimulation in galactose was more affected in HBV-specific than CMV-specific responses (Figure 3C).

Next, we asked whether there was an association between Glut1 expression and the ability of virus-specific cells to mediate effector function in the absence of glucose. Therefore, we split our cohort into patients for whom the response was more severely affected (reduction in IFNγ frequency below the mean of the cohort; gray shaded area in Figure 3B) and those for whom the response remained above the mean of the cohort. HBV-specific responses most affected by blocking glycolysis had significantly more Glut1^+^ cells then those that were less affected, while a non-significant difference was observed for CMV-specific cells (Figure S3B). These data suggest that the upregulation of Glut1 reflects a dependence on glycolysis in exhausted T cells.

The finding that HBV-specific T cells were unable to utilize OXPHOS for effector function prompted us to investigate whether they had a mitochondrial defect. Mitochondrial mass (MM) can be assessed by staining cells with the mitochondrial-potential-independent dye MitoTracker green, previously used to assess the presence of enlarged non-functional mitochondria in T cells (Henson et al., 2014). HBV-specific CD8 showed a higher MM compared to CMV-specific CD8 from the same donors (Figure 3D). The increased MM in HBV- compared to CMV-specific CD8 was also apparent ex vivo (Figure 3E).

We then used the ratiometric fluorescent dye JC-1 to assess mitochondrial potential, a key readout of mitochondrial function. HBV-specific T cells showed a much lower mitochondrial potential than CMV-specific cells, as indicated by the drop in the red/green fluorescence intensity ratio of the probe, which is independent of mitochondrial size or shape (Figure 3F). A decrease in mitochondrial potential could be a sign of apoptosis induction; however, we found HBV- and CMV-specific T cells to be viable, as they were negative for dead-cell staining and showed low annexin V staining, comparable to global T cells in the same culture (Figure S3C). This indicates that mitochondrial dysfunction in HBV-specific T cells limits their capacity to fuel their energy demand for cytokine production by switching to OXPHOS.

### The Third Signal Cytokine IL-12 Enhances the Metabolic Function of Exhausted HBV-Specific T Cells

Recently, we demonstrated that the third signal pro-inflammatory cytokine IL-12 enhanced effector responses in exhausted HBV-specific T cells, while it had little effect on the functional CMV-specific response. When HBV-specific T cells were stimulated with viral peptide in the presence of IL-12, IFNγ production, TNF production, and cytotoxicity were significantly increased (Schurich et al., 2013). We postulated that the capacity of IL-12 to reconstitute HBV-specific T cell effector function might be mediated by a change in the efficiency of their glucose metabolism.

**Figure 4:**
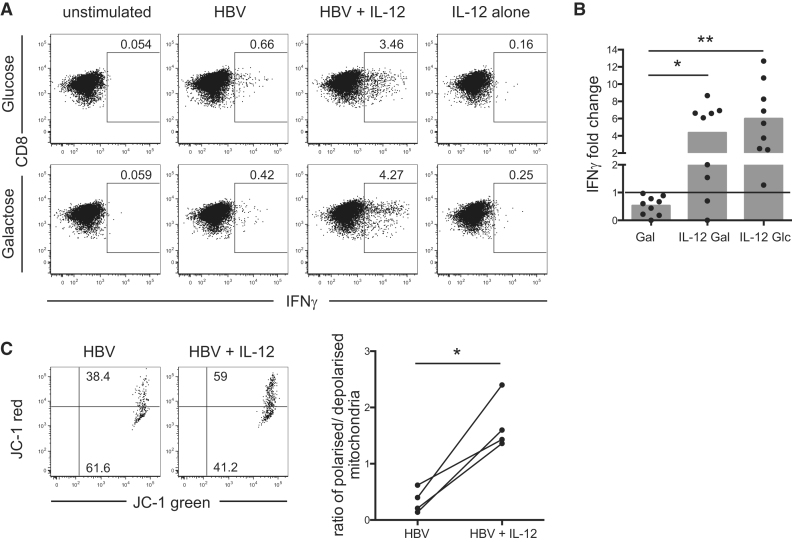
Stimulation with the Pro-inflammatory Cytokine IL-12 Recovers HBV-Specific Responses even when Glycolysis Is Suppressed (A) PBMCs were stimulated with HBV-derived peptides in the presence or absence of IL-12, cultured for 9 days, and transferred into media containing either 10 mM glucose or 10 mM galactose for 24 hr before peptide restimulation for functional analysis. Representative FACS plots of the percentage of IFNγ+ CD8 cells when cultured in glucose or galactose and stimulated with HBV peptides, with or without IL-12, or IL-12 alone. (B) Summary data showing the IFNγ response to HBV peptides in galactose alone, galactose with IL-12, or glucose with IL-12, plotted as the fold change compared to the response of cells stimulated in glucose alone (set to 1, indicated as line in the graph). The mean response is indicated with gray bars, and individual responses are indicated with dots. (C) Change in mitochondrial polarization upon stimulation of HBV-specific T cells with IL-12, representative staining with JC-1 (left), and summary data comparing the ratio of polarized to depolarized mitochondria (right). ^∗^p < 0.05; ^∗∗^p < 0.005.

To test this, we stimulated and cultured T cells in the presence or absence of IL-12 and restimulated in the presence of either glucose or galactose. When IL-12 enhanced IFNγ production by HBV-specific T cells, the cytokine did so regardless of whether the cells were restimulated in glucose or galactose (Figures 4A and 4B). Furthermore, IL-12-stimulated HBV-specific CD8 had a significantly increased ratio of polarized, compared to depolarized, mitochondria (Figure 4C). These findings indicate that IL-12 can stimulate mitochondrial and metabolic changes in exhausted HBV-specific T cells, reversing their dependence on glycolysis for effector function.

## Discussion

Glycolysis, accompanied by the upregulation of the glucose transporter Glut1, has been described to be the main metabolic pathway fueling effector function upon T cell activation (MacIver et al., 2013). Surprisingly, we found that exhausted HBV-specific T cells, which have very limited effector capacity, showed a marked increase in expression of functional Glut1 upon antigenic stimulation, suggesting that their impaired effector function was not due to a lack of glucose uptake. In contrast to HBV-specific CD8, analysis of paired samples revealed that the more functional CMV-specific CD8 expressed less Glut1, despite equivalent levels of antigenic reactivation. This implied that HBV- and CMV-specific T cells might utilize different metabolic pathways. We tested this hypothesis by culturing the cells in galactose, which impairs glycolysis, to probe the capacity of cells to use the alternative pathway of generating energy via mitochondrial OXPHOS (Chang et al., 2013). We found that HBV- and CMV-specific T cells are differentially affected by this method of favoring OXPHOS over glycolysis. CMV-specific CD8 T cells from many patients produced normal, or even elevated, amounts of effector cytokines upon culture in galactose, while, in others, the production declined but was never completely abrogated. These data do not contradict previous findings regarding the importance of glycolysis for IFNγ production in CD4 T cells (Chang et al., 2013). In HBV-specific cells, effector cytokine production declined or was completely lost. Defects in the glycolytic pathway cannot be formally excluded, but our data indicate that exhausted HBV-specific T cells rely on generating their energy through glycolysis and cannot compensate by using other pathways. Our findings indicate that, in some virus-specific effector T cells, mitochondrial respiration is used to supplement glycolysis in order to satisfy the demands of efficient cytokine production, in line with increased proliferation, survival, and anti-viral function in CD8 T cells with genetically enhanced OXPHOS (Okoye et al., 2015). The generation of reactive oxygen species (ROS) is vital in the activation of CD4 T cells (Sena et al., 2013), and an inability of HBV-specific T cells to use OXPHOS could partially be due to changes in ROS production. However, we did not find a significant difference in the production of ROS in exhausted, compared to functional, CD8 (data not shown).

The observed inability of HBV-specific CD8 T cells to use OXPHOS to fuel their effector function pointed to a mitochondrial defect. This was supported by their increased mitochondrial depolarization compared to that of CMV-specific T cells, indicative of impaired function. Additionally, an increase in MM in HBV-specific, compared to CMV-specific, CD8 T cells could be due to the formation of non-functional giant mitochondria, as have been described to accumulate in terminally differentiated human CD8 T cells (Henson et al., 2014).

We found that the most exhausted T cells, as measured by decrease in cytokine production and increase in co-inhibitory PD-1, showed the highest expression of Glut1 and the lowest ability to maintain effector functions when forced to use OXPHOS. Our data, therefore, reveal that functional exhaustion is linked to metabolic impairments in human-virus-specific CD8 T cells. This link is underscored by the impact of IL-12; its capacity to rescue HBV-specific CD8 T cells from exhaustion and enhance their functionality (Schurich et al., 2013) is paralleled by an increased proportion of cells harboring polarized mitochondria and a reduction in their dependence on glycolysis. Of note, we have previously shown that IL-12 can reduce PD-1 expression on CD8 T cells (Schurich et al., 2013), and the contribution of this pathway to the metabolic changes observed is currently under investigation.

Global patient-derived PD-1+ CD8 T cells had a low SRC, suggesting that their ability to generate additional energy through oxygen consumption in situations of metabolic stress is reduced. PD-1 signaling has previously been described to reduce glycolysis and promote fatty acid oxidation in global CD4 T cells (Patsoukis et al., 2015), but the impact of PD-1 signaling on the metabolism of exhausted CD8 cells remains to be investigated. It is also important to keep in mind that T cells in patients with chronic HBV express additional inhibitory molecules like Tim-3 and CTLA-4 (Nebbia et al., 2012, Schurich et al., 2011); therefore, multiple signals might shape the altered metabolism in these cells.

It will be interesting to establish whether exhausted T cells in other settings (HIV or HCV infection, tumors) have a similar deficiency in their capacity to supplement glycolysis with OXPHOS in order to optimize their energy supply. The mitochondrial defects and adaptation to glycolysis that we documented here may be the result of particular features of chronic HBV, such as high-level antigenic stimulation in the immunosuppressive liver environment. In particular, the hypoxic milieu of the liver may be relevant since hypoxia-inducible factor (HIF)-1α/β induces the transcription of Glut1 and multiple rate-limiting glycolytic enzymes to sustain glycolysis in activated T cells (Finlay et al., 2012). We found that intrahepatic T cells showed an increased expression of Glut1 ex vivo and that hypoxia during antigenic stimulation in vitro could recapitulate this upregulation. The liver can produce IL-7 in response to TLR signaling, thereby enhancing T cell survival and function (Sawa et al., 2009); it is plausible that this could be partially mediated by the capacity of IL-7 to drive glycolysis through the induction of Glut1 (Loisel-Meyer et al., 2012, Wofford et al., 2008). The metabolic phenotype of HBV-specific T cells could, therefore, be a consequence of, or an adaptation to, their target environment, allowing the few remaining responses to survive.

In summary, our data show that different virus-specific T cells in the same host have contrasting usage of pathways for glucose metabolism. The exhausted T cell response directed against HBV is characterized by an upregulation of the glucose transporter Glut1, a dependency on glycolysis, and mitochondrial defects in OXPHOS and depolarization. In contrast, the highly functional CMV-specific T cell response can utilize OXPHOS for effector function when glycolysis is blocked. Our finding that a more protective T cell response requires mitochondrial respiration is consistent with findings from two recent papers emphasizing the importance of OXPHOS for antigen-specific T cell expansion and antiviral immunity in mice (Okoye et al., 2015, Sena et al., 2013). A better understanding of why this pathway fails in exhausted T cells may reveal mitochondrial targets for therapeutic boosting of antiviral immunity.

## Experimental Procedures

### Patients

#### Ethics Statement

This study was approved by the ethical boards of the Royal Free Hospital and Barts and The London NHS trust, and written informed consent was obtained from all participants. All participants were HCV and HIV seronegative and HBV treatment naive. HLA-A2 status was determined by specific antibody (AbD Serotec). Patient information is provided in Table S1.

### Overnight and Short-Term Cell Culture and Stimulation

PBMCs were isolated by Ficoll-Hypaque density gradient centrifugation and either analyzed directly or cryopreserved. Liver biopsy sections, surplus to diagnostic requirements, were homogenized and filtered to obtain intrahepatic lymphocytes. To examine virus-specific T cell responses, PBMCs from HLA-A2^+^ donors were stimulated with 1 μM HBV-derived HLA-A2 restricted peptides (core, FLPSDFFPSV; envelope, FLLTRILTI, WLSLLVPFV, LLVPFVQWFV, and GLSPTVWLSV; polymerase, GLSRYVARL and KLHLYSHPI) (Proimmune) or stained with dextramers loaded with the aforementioned peptides (Immudex). If derived from HLA-A2^−^ donors, PBMCs were stimulated with 1 μg overlapping peptides spanning the whole HBV core protein, with sequence correlating to HBV genotype D (AYW) (JPT Peptide Technologies). Responses to CMV were measured using 1 μM NLVPMVATV peptide (Proimmune) or dextramers (Immudex) for HLA-A2^+^ donors or overlapping pp65 for HLA-A2^−^ donors (JPT Peptide Technologies). All cultures were supplemented with 20 U/ml rhIL-2 (Miltenyi Biotech) at days 0 and 4 and with the addition of rhIL-12 (Miltenyi Biotech) at 10 ng/ml at day 0 where indicated. PBMCs were restimulated on day 9 for 4 hr by re-adding peptide at the original concentration in the presence of 1 μg/ml brefeldin A (BFA; Sigma-Aldrich). To assess metabolic requirements, samples were split and transferred into media containing either 10 mM glucose or 10 mM galactose (supplemented with 1 mM sodium pyruvate) on day 8 for 24 hr; peptide and BFA were added for the final 4 hr. Virus-specific responses were identified by IFNγ or TNFα production.

### Impact of Hypoxia

PBMCs were cultured and peptide stimulated as described earlier at normoxia (21% 0_2_). At the end of culture, PBMCs were split and peptide restimulated in the presence of BFA overnight at 5% O_2_ (hypoxia) or 21% O_2_ (normoxia). Virus-specific responses were identified by IFNγ.

### Flow Cytometric Analysis

PBMCs were stained for surface markers CD8 (OKT8) (eBiosciences), CD3 (UCHT1) (eBiosciences), PD-1 (EH12.2H7) (Biolegend), and CD38 (HIT2) (BD Biosciences). Dead cells were always excluded using a live/dead fixable dye staining kit (Invitrogen). Cells were fixed and permeabilized for detection of intracellular molecules using anti-IFNγ (B27), TNFα (MAB11) (BD Biosciences), and Glut1 (202915) (RnD Systems). JC-1 (2 μM) (Molecular Probes), MitoTracker Green (100 nM) (Invitrogen) were used per manufacturer’s instructions. Samples were acquired on a BD Fortess, analysis was performed using FlowJo (Tree Star).

### Metabolic Assay

OCR and ECAR were measured on an XF-24 Extracellular Flux Analyzer (Seahorse Bioscience). CD8 T cells were stimulated with 1 μg/ml CD3 (OKT3) (eBiosciences) and 20 U/ml IL-2 in non-buffered RPMI 1640 medium during the assay. Inhibitors were 1 μM oligomycin, 1.5 μM FCCP, 1 μM antimycin A (Sigma Aldrich), and 100 nM rotenone (Seahorse Bioscience).

### Statistical Analysis

Statistical analyses were performed using the non-parametric Mann-Whitney or Wilcoxon matched-pairs test as appropriate, and significant differences are indicated in the figures (^∗^p < 0.05; ^∗∗^p < 0.005; ^∗∗∗^p < 0.0005).

## Author Contributions

Conceptualization, A.S. and M.K.M.; Investigation, A.S., L.J.P., D.J., J.W., and I.O.; Resources, U.S.G., N.H., P.T.K., E.N., R.G., and M.K.M.; Supervision, A.S., C.F., S.M.H., and M.K.M.; Writing – Original Draft, A.S.; Writing – Review and Editing, A.S. and M.K.M.; Funding Acquisition, M.K.M.

## Supplemental Information

Document S1. Figures S1–S3 and Table S1

Document S2. Article plus Supplemental Information
