# Supplementary material for: Distinct Metabolic Requirements of Exhausted and Functional Virus-Specific CD8 T Cells in the Same Host
Source: Cell Rep. 2016 Jul 21;16(5):1243–52. doi: 10.1016/j.celrep.2016.06.078 (PMC4977274; doi:10.1016/j.celrep.2016.06.078)
Supplement: Document S1. Figures S1–S3 and Table S1 [file mmc1.pdf]

Cell Reports, Volume *16*

Supplemental Information

Distinct Metabolic Requirements of Exhausted and Functional Virus-Specific CD8 T Cells in the Same Host

Anna Schurich, Laura J. Pallett, Danyal Jajbhay, Jessica Wijngaarden, Itziar Otano, Upkar S. Gill, Navjyot Hansi, Patrick T. Kennedy, Eleni Nastouli, Richard Gilson, Christian Frezza, Sian M. Henson, and Mala K. Maini

Supplementary Figure 1

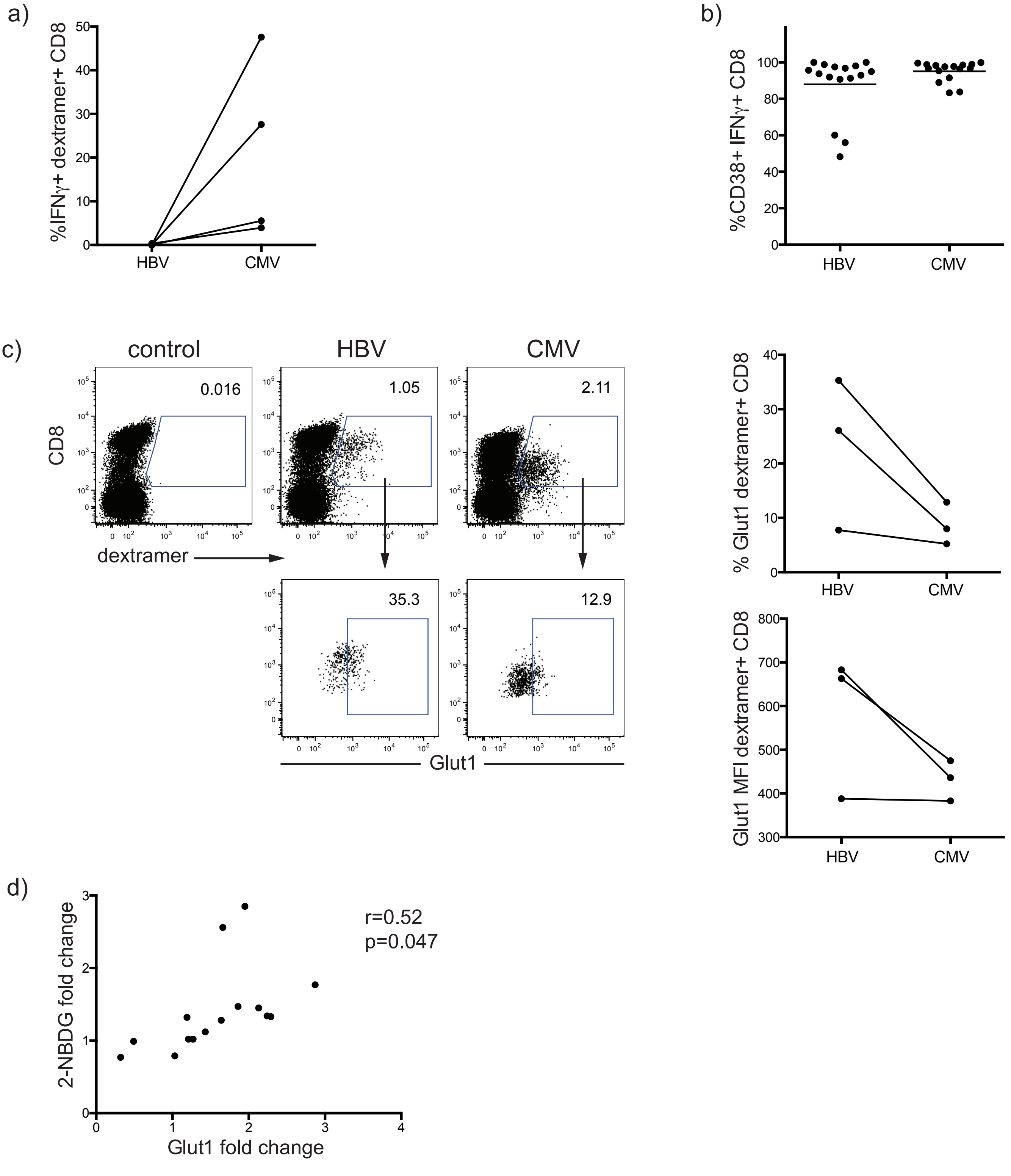

Supplementary Figure 2

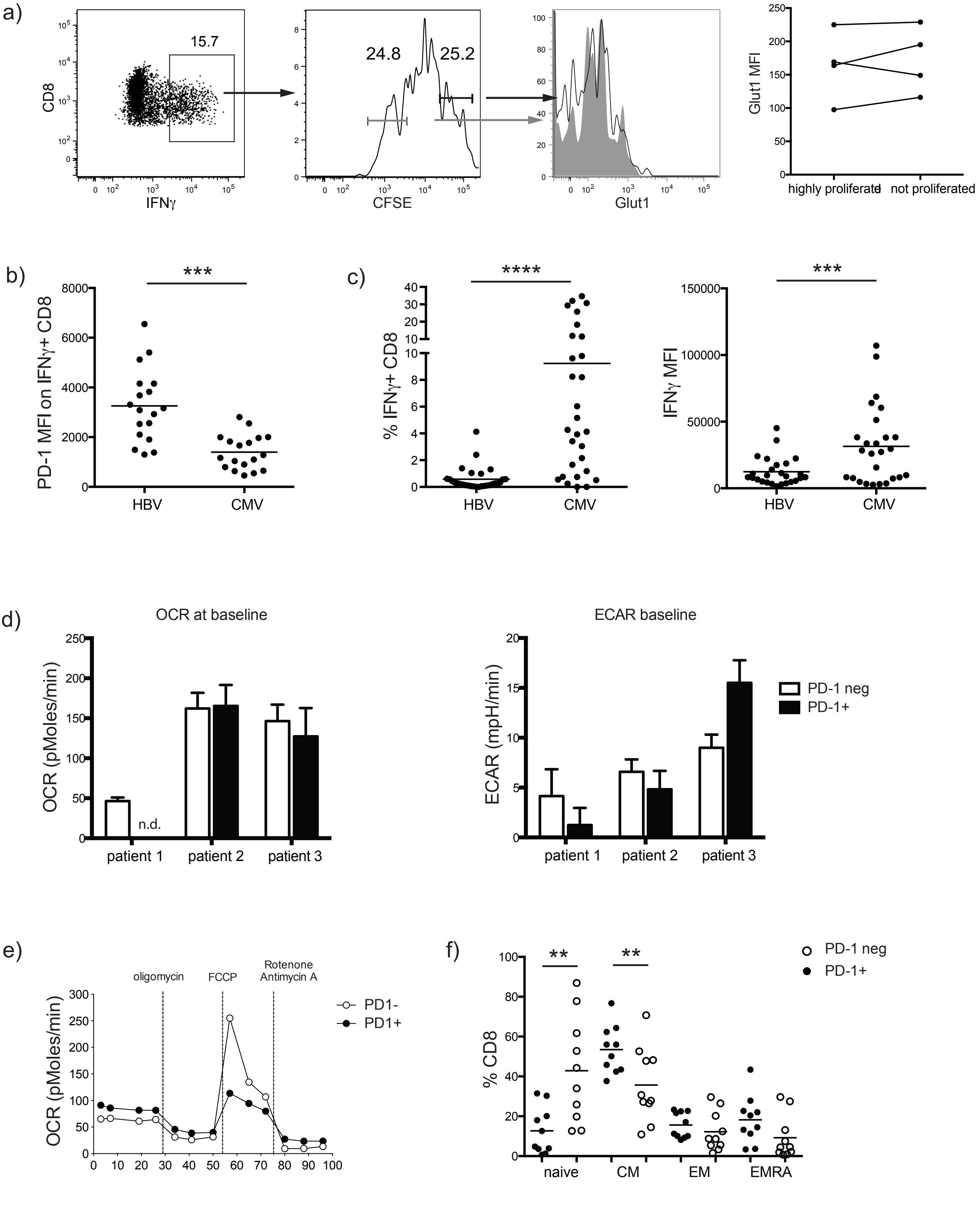

Supplementary Figure 3

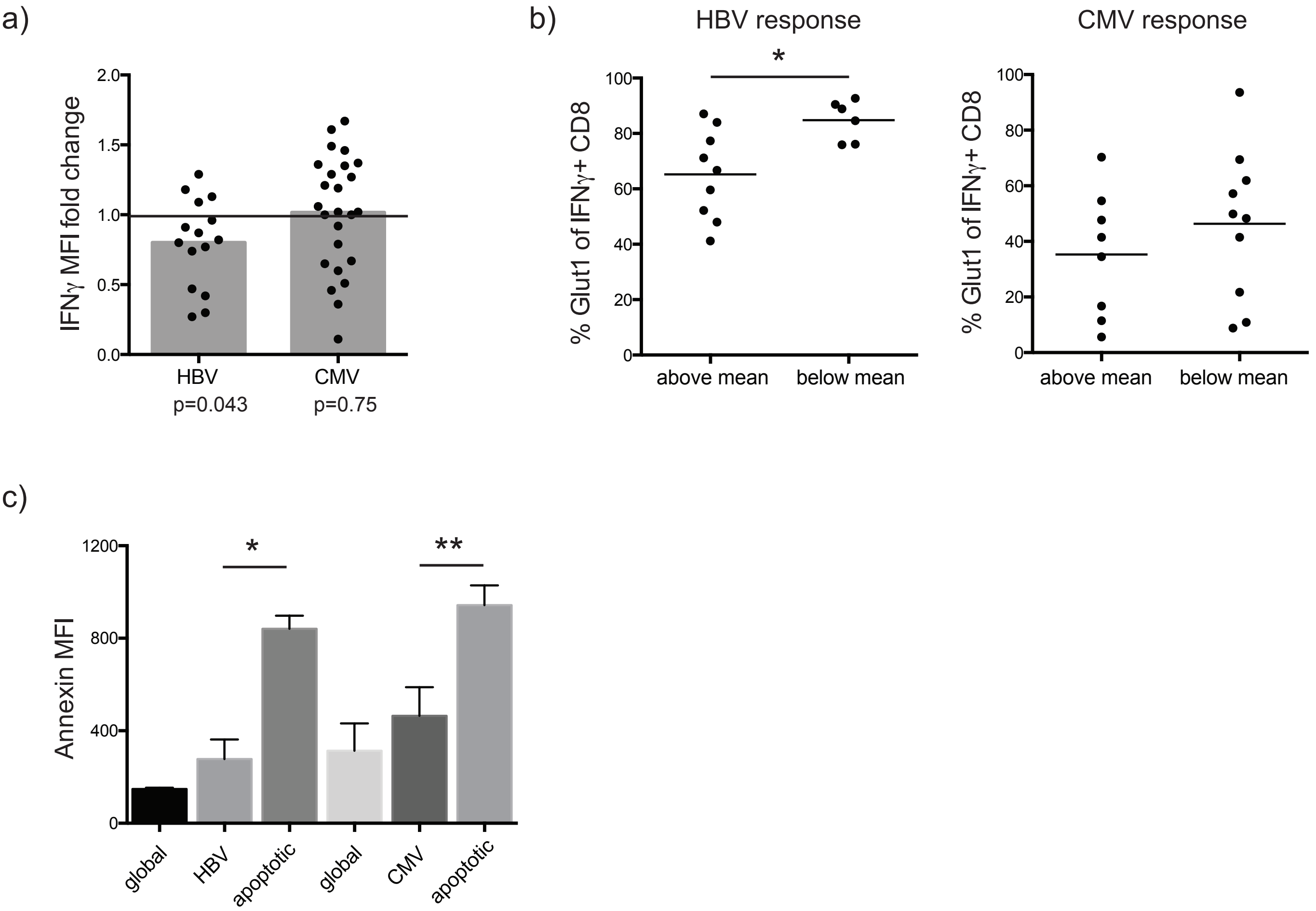

Supplementary Figure 1, related to Figure 1: HBV and CMV-specific T cells retain distinct phenotypes in culture

Summary data of paired HBV-and CMV-specific T cells from patients with CHB detected ex vivo through HLA-A2 dextramer staining and then stimulated for 4hrs with cognate peptide to induce IFNγ production (a) and after 10day culture: activation status assessed by %CD38 expression of IFNγ$^+$ CD8 (b). Glut1 expression in virus-specific CD8 T cells, detected through HLA-A2 dextramer staining (c). Correlation of the fold change in HBV compared to CMV mediated glucose uptake with the fold change in Glut1 expression between the two subsets (d).

Supplementary Figure 2, related to Figure 2: Metabolic profile of patient-derived PD-1$^+$ and PD-1 negative CD8 T cells ex vivo and after culture

Glut1 expression is independent of proliferation, representative CMV-response (left) and CFSE profile (cells divided over night in 5% oxygen) (middle), Glut1 expression is compared in the 25% most highly divided (grey shaded) and 25% least divided (black line) cells and summary (far right) (a). Expression of the co-inhibitory receptor PD-1 on IFNγ$^+$ CD8 (b) and frequency (%) and amount of IFNγ produced (mean fluorescence intensity MFI) by CD8 (c). Comparison of PD-1$^+$ and PD-1$^-$ CD8 T cells stimulated with anti-CD3 and IL-2 *ex vivo.* Oxygen consumption rate (OCR) and extracellular acidification rate (ECAR) were measured in real time (mean of 4 base line readings shown) (d). Example of metabolic profile of PD-1$^+$ and PD-1 negative CD8 T cells after 10day culture *in vitro*. Cells were stimulated with anti-CD3 and IL-2 during measurement of OCR and mitochondrial inhibitors added as indicated (e). Differentiation status of

global PD-1+ and PD-1 negative CD8 T cells in chronic HBV defined as naïve: CD45RA+, CD27+, central memory (CM) CD45RA-, CD27+, effector memory (EM) CD45RA-, CD27- and terminally differentiated (EMRA) CD45RA+, CD27- (f).

Supplementary Figure 3, related to Figure 3: CD8 expressing high Glut1 are the most dependent on glycolysis.

(a) Summary data showing the IFNγ MFI of the virus-specific response upon restimulation in galactose, plotted as fold change compared to response in glucose (set to one as indicated by line in the graph). The mean response is shown as grey bars and individual responses as dots. (b) Glut1 expression in IFNγ^{+} HBV-specific T cells (left panel) or CMV-specific T cells (right panel), divided according to whether the response to culture galactose is above or below the mean (all samples falling in grey shaded area in Fig3b left panel). (c) Staining with the apoptosis marker Annexin V in global, virus-specific and apoptotic dead cells within the same wells (n=6). (d) Model of metabolism in functional CMV-specific compared to exhausted HBV-specific CD8 T cells.

Supplementary table 1, related to materials and methods

	Number	Gender (m)	Age	HBV DNA (IU/ml)	ALT (U/L)	eAg+
**CHB patients**	132	77	35 (18-64)	825 (1-300,000,000)	31 (10-250)	16

Patient characteristics. Data shown is the median value and spread from lowest to highest values in brackets. In cases where viral load was below quantification the value was set to 1.
